# Transcriptome analysis reveals the promoting effects of exogenous melatonin on the selenium uptake in grape under selenium stress

**DOI:** 10.3389/fpls.2024.1447451

**Published:** 2024-08-22

**Authors:** Jin Wang, Yuhang Lu, Shanshan Xing, Jinman Yang, Lei Liu, Kewen Huang, Dong Liang, Hui Xia, Xiaoli Zhang, Xiulan Lv, Lijin Lin

**Affiliations:** ^1^ College of Horticulture, Sichuan Agricultural University, Chengdu, China; ^2^ Institute of Horticulture Chengdu Academy of Agriculture and Forestry Sciences, Chengdu, China

**Keywords:** grape, growth, melatonin, selenium, transcriptome

## Abstract

**Introduction:**

Exogenous melatonin (MT) can promote horticultural crops growth under stress conditions.

**Methods:**

In this study, the effects of exogenous MT on the accumulation of selenium (Se) in grape were studied under Se stress.

**Results and discussion:**

Under Se stress, exogenous MT increased the biomass, content of photosynthetic pigments and antioxidant enzyme activity of grapevines. Compared with Se treatment, MT increased the root biomass, shoot biomass, chlorophyll *a* content, chlorophyll *b* content, carotenoids, superoxide dismutase activity, and peroxidase activity by 18.11%, 7.71%, 25.70%, 25.00%, 25.93%, 5.73%, and 9.41%, respectively. Additionally, MT increased the contents of gibberellin, auxin, and MT in grapevines under Se stress, while it decreased the content of abscisic acid. MT increased the contents of total Se, organic Se and inorganic Se in grapevines. Compared with Se treatment, MT increased the contents of total Se in the roots and shoots by 48.82% and 135.66%, respectively. A transcriptome sequencing analysis revealed that MT primarily regulated the cellular, metabolic, and bioregulatory processes of grapevine under Se stress, and the differentially expressed genes (DEGs) were primarily enriched in pathways, such as aminoacyl-tRNA biosynthesis, spliceosome, and flavonoid biosynthesis. These involved nine DEGs and nine metabolic pathways in total. Moreover, a field experiment showed that MT increased the content of Se in grapes and improved their quality. Therefore, MT can alleviate the stress of Se in grapevines and promote their growth and the accumulation of Se.

## 1 Introduction

Selenium (Se) is a beneficial element for the humans, and participates in at least three major metabolic processes necessary for cellular metabolism of humans (Rayman, 2012; Roman et al., 2014). Thus, it is necessary for humans to consume crops enriched with Se to obtain supplemental amounts of this element. To alleviate the deficiency of Se in the soil and improve its accumulation in crops throughout the world, the continuous use or over-application of Se fertilizers for biofortification has increased its accumulation in crops. This substantially contributes to the release of Se from agroecosystems, which leads to the environmental Se contamination and potential toxicity of Se on crops (Mostofa et al., 2017). So, it is necessary to implement measures to alleviate stress in crops caused by Se and increase Se uptake in crops.

Melatonin (MT) is a small molecule of the indole class of tryptamines that is found widely in nature, distributed in various organs and involved in circadian rhythms in plants, seed germination, root development, fruit ripening, and photosynthesis among others (Arnao and Hernández-Ruiz, 2015; Arnao and Hernández-Ruiz, 2020). Studies have shown that MT functions as a direct scavenger or induces the antioxidant system to indirectly scavenge reactive oxygen species (ROS) in plants, which helps to increase their resistance to oxidative stress (Rodriguez et al., 2004; Sliwinski et al., 2007). In *Arabidopsis thaliana*, several transcription factors (TF) involved in the plant stress response are induced by MT, including zinc finger protein 6 (ZAT6), AXR3/IAA17 proteins, heat shock factor 1s (HSFA1s), CBFs-binding factors, and drought response element-binding factors 1 (DREB1s) (Shi and Chan, 2014; Shi et al., 2015a; Shi et al., 2015b). Under drought stress, the application of MT enhances photosynthesis in kiwi seedlings by inhibiting stomatal closure, enhancing the uptake of light energy, promoting electron transport in Photosystem II, and improving the fixation of carbon dioxide (CO_2_) (Liang et al., 2019). Under low-temperature stress, MT can also reduce the contents of malondialdehyde and relative conductivity in grape, thereby enhancing their tolerance to cold (Tong et al., 2019). Under heavy metal stress, MT can alleviate the cadmium (Cd) stress on the adverse physiological activity of strawberry (Wu et al., 2021). The application of MT reduces the accumulation of Cd and alleviates its toxicity in apple shoots by increasing the levels of expression of the key genes for detoxification (He et al., 2020). Under Se stress, exogenous MT induces the antioxidant system to reduce the accumulation of ROS in oilseed rape, increases the levels of water and sugars in the leaves to alleviate osmotic stress, and increases the content of thiol chelates in the roots to inhibit the translocation of Se within oilseed rape cells (Ulhassan et al., 2019). Moreover, exogenous MT enhances the nitrogen uptake and its utilization and accumulation in apple by increasing the expression levels of nitrogen uptake related genes (*AMT1;2*, *AMT1;5*, *NRT1;1*, and *NRT2;4*) and its metabolism (*NR*, *NiR*, *GS*, and *Fd-GOGAT*) under moderate drought conditions (Liang et al., 2018). Exogenous MT can also increase the ATP content and the activity of the hydrogen ion (H^+^) pump in rice roots, which enhances the Na^+^ efflux and K^+^ influx from the root cells, thus, maintaining Na^+^/K^+^ homeostasis in the rice tissues under salt stress (Yan et al., 2021). In tomato, the application of MT promotes the uptake and assimilation of S by regulating the expression levels of S transport proteins (SUT1;1 and SUT1;2) related genes and the key enzymes of sulfur (S) metabolism in plants that were deficient in Se (Hasan et al., 2018). These studies have shown that exogenous MT is involved in the uptakes of multi-elements in plants, but there are a few studies related to the accumulation of Se in plants (Liao et al., 2018; Cheng et al., 2022).

Grape is one of delicious fruits with a low content of Se in its berries (Jin et al., 2020; Zhang, 2020). However, an improvement in living standards has led consumers to demand higher quality grapes, and they now emphasize their health functions (Zhang, 2020). Thus, it is highly practically significant to improve the ability of grapes to take up Se in the original soil. Concentrations of 50-200 µmol/L MT have been previously shown to promote the Se uptake by grapevines under Se stress, and the best concentration was determined to be 150 µmol/L (Cheng et al., 2022). However, the action mechanism of MT on the Se accumulation in grapes still remains unknown. In this study, we further examined the effects of exogenous MT on the stress physiology and accumulation of Se in grapevines under Se stress and used transcriptome sequencing to reveal the regulatory mechanism of MT on the accumulation of Se in grapes to determine the ability of MT to regulate the pathways related to the metabolism of Se and the differentially expressed genes (DEGs). Moreover, the effects of exogenous MT on the quality and Se accumulation in grapes were studied using field experiments to determine the effects of applying MT on the enrichment of Se in grapes.

## 2 Materials and methods

### 2.1 Pot experiment design

The variety of grape used in this experiment was ‘Summer Black’ (triploid seedless grape), because the previously study showed that MT promoted its Se uptake. Grape branches were collected from the vineyard of Sichuan Agricultural University (33°33′N, 103°38′E), Chengdu, China, in December 2019 and stored at 4 °C in moist sand. In April 2020, the branches were cut into 10-cm-long pieces with one bud and planted in 50-hole plug trays filled with perlite. Grape seedlings were grown and managed as described by Liu et al. (2021).

In June 2020, when the new shoots of grape seedlings had grown to 20 cm, the uniform plant seedlings were transplanted into 15-cm high plastic pots that were 18 cm in diameter and contained perlite. Each pot contained two seedlings and was placed in a greenhouse with specific conditions. During the day, the greenhouse had 14 h of light at 10,000 Lux, a temperature of 25°C, and a relative humidity of 70%, while at night, it had 10 h of darkness at 0 Lux, a temperature of 20°C, and a relative humidity of 90% (Li et al., 2022). At 7 d, the plants were watered with Hoagland solution every 3 d to ensure that they had fully regrown, and each pot was irrigated with 100 mL each time. Four treatments were then conducted, including (1) no additional Se (CK), (2) no additional Se + MT application (MT), (3) additional Se (Se), and (4) additional Se + MT application (SeMT). Each treatment was repeated three times (two pots as a replicate and 24 pots in total) using a completely randomized design. The Hoagland’s nutrient solution contained 0.1 mg/L Se (in the form of Na_2_SeO_3_) (Liu et al., 2021). A volume of 100 mL of Hoagland’s nutrient solutions that contained or lacked Se were used to irrigate each pot of plants every 3 d until they were harvested. A solution of MT (150 µmol/L) was sprayed on the plants to apply this compound as described by Cheng et al (Cheng et al., 2022). in the evening. The same volumes of deionized water were sprayed on the treatments that were not treated with MT. A solution of MT or deionized water was sprayed every 7 d for a total of four sprays. The plants were harvested one month (July 2020) after the first spray of MT. One pot of each replicate was used to collect the mature fresh leaves to determine their contents of photosynthetic pigments and endogenous phytohormones, activities of antioxidant enzymes and those related to the metabolism of Se activity, and the transcriptome sequencing analysis. The other pots of each replicate were used to determine the biomass and content of Se.

### 2.2 Field experiment design

The variety of grape was ‘Summer Black’ (4-year old), and its rootstock was ‘Beta’. Grape plants were planted in the vineyard of Sichuan Agricultural University. The plants were spaced 1.5 m × 3.0 m with 2,250 plants/ha. There were 0.33 ha of ‘Summer Black’. A rain shelter was used in the field experiment. The vineyard soil was fluvo-aquic, and its basic physical and chemical properties were pH 7.82, organic matter content 24.32 g/kg, total nitrogen content 1.25 g/kg, total phosphorus content 13.26 g/kg, total potassium content 16.53 g/kg, alkali-hydrolyzable nitrogen content 83.24 mg/kg, available phosphorus content 26.33 mg/kg, available potassium content 110.32 mg/kg, and total content of Se 0.01 mg/kg.

When grapes began to change color in June 2020, 36 uniform grape plants were selected for the field experiment. Four treatments were conducted, including (1) no Se supply (CK), (2) no Se supply + MT application (MT), (3) Se supply (Se), and (4) Se supply + MT application (SeMT). Each treatment was repeated three times using a completely randomized design. Three grape plants served as a replicate. The Se was supplied in the form of sodium selenite (Na_2_SeO_3_) for each grape plant that was irrigated in soil to ensure that there was 0.25 mg/kg of Se in the 0-50 cm soil as described by Dinh et al (Dinh et al., 2018). The control that lacked Se was irrigated with the same volume of deionized water. Moreover, a 150 µmol/L solution of MT (dissolved in distilled water) was sprayed on the both sides of grape leaves in the evening. The CK was sprayed with the same volume of deionized water. MT or deionized water was sprayed every 7 d for a total of four sprays. When grapes became commercially mature in July 2020, three to four grapes were collected from the upper, middle and lower parts of each bunch. There were a total of 243-324 grapes for each treatment. The samples of grapes were used to determine the quality indicators and total Se content.

### 2.3 Determination of the physiological parameters and biomass

The first mature leaf from the top was collected to determine the contents of chlorophyll *a*, chlorophyll *b*, and carotenoids by soaking the fresh leaves in a 1:1 mixture of ethanol and acetone (v/v) and determining the absorbance at 663, 645, and 470 nm using a spectrophotometer (Summit, Shanghai, China) (Hao et al., 2004; Lin et al., 2020). The second mature leaf from the top was collected to assay the activities of superoxide dismutase (SOD), peroxidase (POD), and catalase (CAT) by the nitroblue tetrazole method, guaiacol colorimetric method, and potassium permanganate titration method, respectively (Hao et al., 2004; Liu et al., 2021).

The third mature leaf from the top was collected to determine the contents of gibberellic acid 3 (GA3), indole-3-acetic acid (IAA), abscisic acid (ABA) and MT using a high-performance liquid chromatography (Agilent 1260 HPLC system, Agilent Technologies, Santa Clara, California, USA) (Liu, 2018; Xiao et al., 2020).

After that, the whole grapevines were harvested and separated into roots and shoots, cleaned with tap water and deionized water. The roots and shoots were then dried and determined the biomass (dry weight) using an electronic balance (with a precision of 0.001).

### 2.4 Determination of the content of Se and the activities of enzymes related to the metabolism of Se

The dried ground plant samples were digested in a 4:1 solution of nitric acid and perchloric acid, reduced by hydrochloric acid, and measured to determine the total content of Se using a hydride generation-atomic fluorescence spectrometry (AFS-9700, Beijing Haiguang Instrument Co., Ltd., Beijing, China) (Li et al., 2022). The translocation factor (TF) was calculated as the shoot total Se content/root total Se content (Rastmanesh et al., 2010). The finely ground dry plant samples were extracted by HCl, and the content of inorganic Se was determined using a hydride generation-atomic fluorescence spectrometry. The organic Se content was calculated as the total Se content - inorganic Se content (Li et al., 2022).

The fourth mature leaf from the top was collected to assay the activities of the enzymes related to Se metabolism, including glutathione peroxidase (GSH-Px), adenosine 5’-phosphosulfate (APS) reductase, serine acetyltransferase (SAT), and selenocysteine methyltransferase (SMT), using enzyme-linked immunosorbent assay (ELISA) kits (Shanghai Enzyme Link Biotechnology Co., Ltd., Shanghai, China) according to the manufacturer’s instructions.

### 2.5 Transcriptome sequencing analysis

The fourth mature leaf from top with three biological replicates was collected for transcriptome sequencing. The RNA was extracted using an RNAprep Pure Plant Kit (TianGen, Beijing, China). The integrity of the extracted RNA was assessed using an Agilent 2100 (Agilent Technologies) according to the manufacturer’s instructions. The RNA sequencing libraries were then generated using an Illumina HiSeq 2500 (Illumina Trading Co., Ltd., Shanghai, China) according to the manufacturer’s instructions.

After sequencing, the acquired raw data (raw reads) were processed, and their sequences were compared against grape reference genome (Kim et al., 2015) using the using the Hisat2 tools. A total of 83.89 Gb clean data was obtained in 12 samples, with 5.89 Gb of clean data for each sample. RSEM software was used to calculate the FPKM value for each sample, while the CD-HIT program was used to eliminate any duplicate consensus reads to generate the final transcripts for further analysis. A subsequent differential expression analysis between the treatments was performed utilizing DESeq2. Genes that exhibited an adjusted *P*-value of < 0.01 and a fold change of ≥ 2 were considered to be differentially expressed. Unigenes were then individually assembled for each sample data using Trinity software. The final unigene sequences were integrated with the COG, GO, KEGG, KOG, NR, Pfam, Swiss-Prot, and EggNOG databases, and unigene annotation information was assessed through comparison using Blast software. Enrichment analyses for the Gene Ontology (GO) and the Kyoto Encyclopedia of Genes and Genomes (KEGG) were performed for the DEGs.

### 2.6 Candidate DEG expression analysis by real-time quantitative reverse transcription PCR

The relative levels of expression of the selected DEGs were determined by qRT-PCR. The primers were designed using Primer Premier 5.0 software based on the reference gene sequences in grape (**Table 1**). *N-VIT_11s0103g00250* served as the internal reference gene. The relative levels of expression of the DEGs were analyzed by the 2^-ΔΔCT^ method (Livak and Schmittgen, 2001).

**Table 1:** Primer information of qRT-PCR.

Gene number	Gene name (ID)	Sequence (5’to3’)
1	*VIT_13s0067g02360*	F: GGTCTCGTGTGCTGATGTT R: CGTCTCTTCGCCCAAGTTT
2	*VIT_12s0035g01160*	F: ATGCTCCTGATGATTACA R: ATCCTTCACACCATACTT
3	*VIT_06s0004g01430*	F: GGAAGTAACGGAAGCATAG R: AGACCACGAGATTGAGAA
4	*VIT_15s0046g01570*	F: GCAGAATACCTATGGAACAAT R: GCCTCCCTCTATGTCAAA
5	*VIT_17s0000g02480*	F: CCAACGGCGACGGCAAGA R: TCATCTTACGGCTCATC TCCTCGG
6	*VIT_19s0027g01190*	F: CTCTTCTTCTGCTCACAA R: ATCATCTCTGCCAATCTT
7	*VIT_19s0093g00220*	F: TAGTGACCCATACCAGAGA R: GACCATACCTTCCTTCCAA
8	*VIT_00s0361g00040*	F: TGCTGTTACCATCAATCA R: GGAAGTCAAGAACTCAATATC
9	*VIT_05s0136g00260*	F: GCATCAAGGACTGGAACT R: GTGTGGAGCGTAACTTCT
10	*N-VIT_11s0103g00250*	F: AGATTACTCCTTGACATTG R: AAGCATTGATTCTGAGATAT

### 2.7 Determination of the quality and total content of Se of grapes

Fresh grapes were used to determine their single weight and the contents of total soluble solids, titratable acid, and vitamin C. Randomly selected batches of 10 single grapes were weighed on an electronic balance. The content of total soluble solids was determined by a hand-held refractometer. The content of titratable acid was determined using the NaOH titration method (Bai, 2003) and calculated by the ratio of the content of total soluble solids to the content of titratable acid. The content of vitamin C was determined by 2,6-dichloroindophenol titration (Bai, 2003). Grapes were dried to determine the content of total Se, which was determined as described for grapevines using a hydride generation-atomic fluorescence spectrometry.

### 2.8 Statistical analysis

SPSS 20.0 (IBM, Inc., Armonk, NY, USA) was used for all the statistical analyses. All the data were normalized and subjected to homogeneity test before analysis using a one-way analysis of variance (ANOVA) and Duncan’s Multiple Range Test (*P <* 0.05). A Pearson correlation was used to analyze the relationships between all parameters in the pot experiment, and the correlation result was visualized using Cytoscape v. 3.5.1 (Cytoscape Consortium, New York, USA). The grey correlations of the different parameters with the content of total Se in the shoots in the pot experiment were analyzed by the grey relational analysis method (Lin et al., 2023; Zhang et al., 2022). All the data in the pot experiment were also subjected to a principal component analysis (PCA) and cluster analysis.

## 3 Results

### 3.1 Biomass and physiological parameters of grapevine

**Figure 1:**
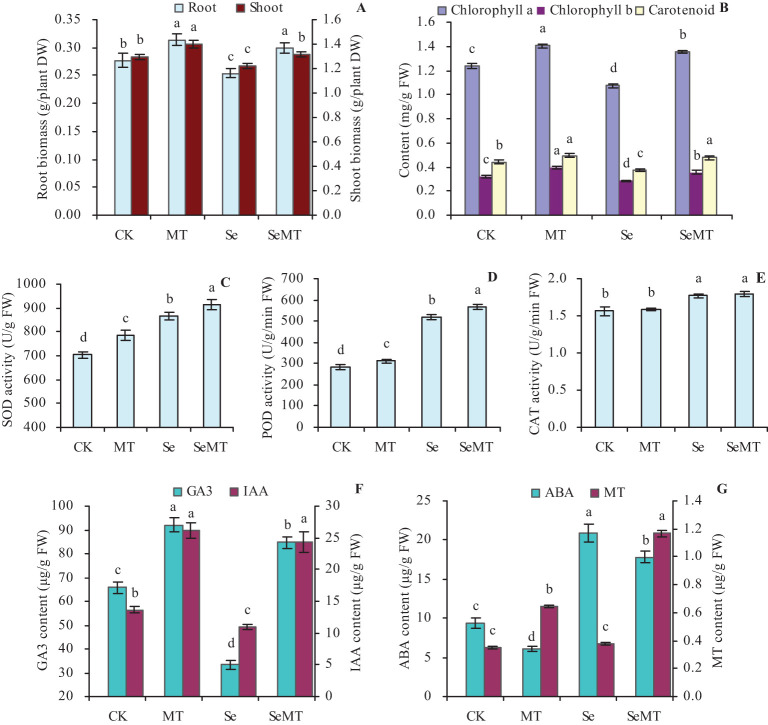
Biomass and physiological parameters of grapevine. **(A)** biomass; **(B)** contents of photosynthetic pigments; **(C)** SOD activity; **(D)** POD activity; **(E)** CAT activity; **(F)** contents of GA3 and IAA; **(G)** contents of ABA and MT. Values are means (± SE) of three replicates. Different letters indicate significant differences among the treatments (Duncan’s Multiple Range Test, *P* < 0.05). DW, dry weight; FW, fresh weight.

Compared with CK, MT treatment increased the biomass and photosynthetic pigment contents of grapevines, which were decreased by the Se treatment (**Figures 1A, B**). Thus, the Se treatment stressed grapevines. SeMT treatment increased the biomass and photosynthetic pigment contents compared with Se treatment and also increased these parameters (except for the shoot biomass) compared with CK. Compared with Se treatment, MT increased the root biomass, shoot biomass, chlorophyll *a* content, chlorophyll *b* content, and carotenoids by 18.11%, 7.71%, 25.70%, 25.00%, and 25.93%, respectively. The different treatments increased the activities of SOD and POD in the order of SeMT > Se > MT > CK (**Figures 1C, D**). Compared with Se treatment, MT increased the activities of SOD and POD by 5.73% and 9.41%, respectively. There were no significant effects on the activity of CAT in MT vs CK and SeMT vs Se treatments, while SeMT treatment increased its activity compared with CK (**Figure 1E**). Compared with CK, MT treatment increased the contents of GA3, IAA, and MT, while Se treatment decreased these values to some extent (**Figures 1F, G**). SeMT treatment also increased the contents of GA3, IAA, and MT compared with Se treatment or CK. However, there was less ABA in the plants treated with MT, while there was more in Se treatment, which was higher than that of CK (**Figure 1G**). There was less ABA in the plants treated with SeMT than Se, while there was more in CK.

### 3.2 Se uptake in grapevines

**Figure 2:**
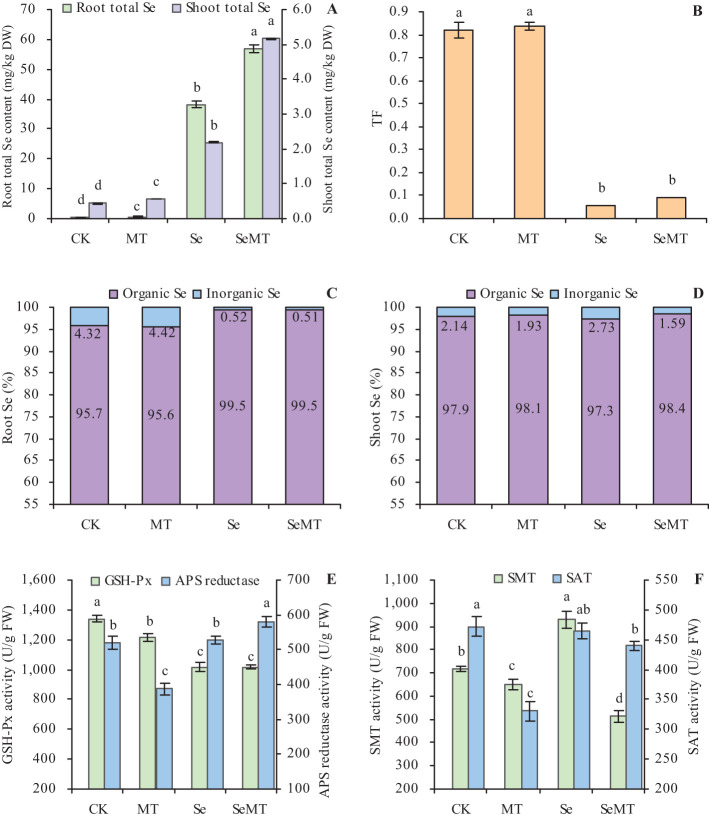
Se uptake in grapevine. **(A)** content of total Se; **(B)** translocation factor (TF); **(C)** ratios of organic Se and inorganic Se to total Se in roots; **(D)** ratios of organic Se and inorganic Se to total Se in shoots; **(E)** activities of glutathione peroxidase (GSH-Px) and adenosine 5’-phosphosulfate (APS) reductase activity; **(F)** activities of selenocysteine methyltransferase (SMT) and serine acetyltransferase (SAT). Values are means (± SE) of three replicates. Different letters indicate significant differences among the treatments (Duncan’s Multiple Range Test, *P* < 0.05). DW, dry weight; FW, fresh weight.

Treatment with MT increased the contents of total Se in grapevines in plants that were treated or not treated with Se (**Figure 2A**). Compared with Se treatment, SeMT treatment increased the contents of total Se in the roots and shoots by 48.82% and 135.66%, respectively. However, MT treatment had no significant effect on the TF of grapevines that were treated and not treated with Se (**Figure 2B**). Treatment with MT also increased the contents of inorganic Se and organic Se in the roots and shoots when plants were treated or not treated with Se (**Table 2**). Treatment with MT decreased the ratio of inorganic Se and increased that of organic Se to total Se in roots, while it had no obvious effects on the ratios of organic Se and inorganic Se to the total Se in shoots (**Figures 2C, D**). In addition, when plants were not treated with Se, MT treatment decreased the activities of the enzymes related to Se metabolism, including GSH-Px, APS, SMT, and SAT (**Figures 2E, F**). Compared with CK, Se treatment decreased the activity of GSH-Px, increased that of SMT, and had no significant effect on the activities of APS and SAT. Treatment of the plants that were enriched with Se with MT increased the activity of APS, decreased that of SMT, and had no significant effect on the activities of GSH-Px and SAT.

**Table 2:** Inorganic Se and organic Se contents in grapevine.

Treatment	Root inorganic Se (mg/kg DW)	Shoot inorganic Se (mg/kg DW)	Root organic Se (mg/kg DW)	Shoot organic Se (mg/kg DW)
CK	0.023 ± 0.002d	0.010 ± 0.000d	0.516 ± 0.015d	0.433 ± 0.020d
MT	0.029 ± 0.001c	0.011 ± 0.000c	0.625 ± 0.012c	0.538 ± 0.004c
Se	0.197 ± 0.004b	0.060 ± 0.002b	38.03 ± 0.944b	2.134 ± 0.010b
SeMT	0.293 ± 0.008a	0.082 ± 0.003a	56.59 ± 1.476a	5.086 ± 0.026a

Values are means (± SE) of three replicates. Different letters indicate significant differences among the treatments (Duncan’s Multiple Range Test, P < 0.05). DW, dry weight.

### 3.3 The DEGs following treatments in grapevine

**Figure 3:**
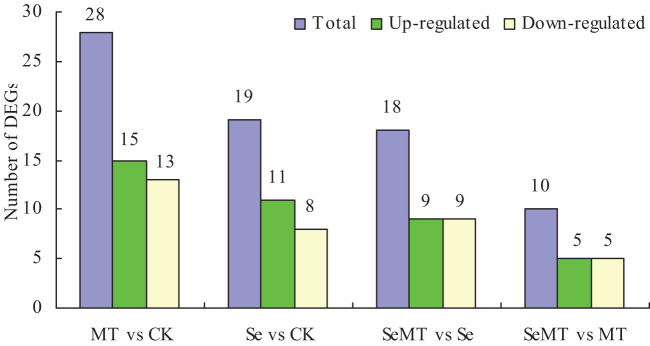
Statistical diagram of DEGs in grapevine.

The sequencing results demonstrated that the GC content exceeded 47% and the Q30 percentage was over 91% (**Table 3**), indicating high quality in sequencing and library construction samples. The sample reads compared with the reference genome had an efficiency ranging from 85.89% to 89.98% (**Table 4**). A comparison of the different treatments showed that a total of 28 DEGs were detected in MT vs CK, and 15 were upregulated and 13 downregulated (**Figure 3**). A total of 19 DEGs were detected in the Se vs CK treatment, and 11 were upregulated and eight downregulated. For the SeMT vs Se treatment, 18 DEGs were detected, including nine upregulated and nine downregulated. There were 10 DEGs for the SeMT vs MT treatment, including five upregulated and five downregulated DEGs. However, there were no shared DEGs in the different comparison groups.

**Table 3:** Statistical table of transcriptome sequencing data.

Samples	Clean reads	Clean bases	GC Content	%≥Q30
CK1	21,660,029	6,425,315,650	48.09%	91.51%
CK2	22,895,958	6,798,891,714	47.71%	91.84%
CK3	19,864,507	5,890,024,016	47.37%	91.75%
MT1	24,082,573	7,165,683,854	47.73%	92.24%
MT2	22,359,875	6,640,499,588	47.60%	91.72%
MT3	21,847,537	6,496,823,200	47.97%	92.57%
Se1	21,371,550	6,355,066,526	47.88%	91.73%
Se2	27,097,299	8,049,154,800	47.58%	92.23%
Se3	25,041,260	7,435,527,720	47.98%	91.94%
SeMT1	26,873,322	7,976,986,036	47.87%	91.73%
SeMT2	22,400,831	6,653,827,062	47.78%	91.68%
SeMT3	26,988,014	8,006,250,566	47.68%	91.73%

**Table 4:** Statistics of sequence comparison of sample sequencing data with the selected reference genome.

Samples	Total Reads	Mapped Reads	Uniq Mapped Reads	Multiple Map Reads	Reads Map to ‘+’	Reads Map to ‘-’
CK1	43,320,058	37,547,996 (86.68%)	36,596,860 (84.48%)	951,136 (2.20%)	18,728,302 (43.23%)	18,748,202 (43.28%)
CK2	45,791,916	40,852,033 (89.21%)	39,818,549 (86.96%)	1,033,484 (2.26%)	20,374,069 (44.49%)	20,393,312 (44.53%)
CK3	39,729,014	35,167,476 (88.52%)	34,293,010 (86.32%)	874,466 (2.20%)	17,536,428 (44.14%)	17,558,403 (44.20%)
MT1	48,165,146	41,847,480 (86.88%)	40,752,994 (84.61%)	1,094,486 (2.27%)	20,879,902 (43.35%)	20,880,695 (43.35%)
MT2	44,719,750	38,466,249 (86.02%)	37,430,589 (83.70%)	1,035,660 (2.32%)	19,166,509 (42.86%)	19,208,476 (42.95%)
MT3	43,695,074	39,314,737 (89.98%)	38,235,571 (87.51%)	1,079,166 (2.47%)	19,628,798 (44.92%)	19,621,974 (44.91%)
Se1	42,743,100	36,890,461 (86.31%)	35,960,388 (84.13%)	930,073 (2.18%)	18,389,404 (43.02%)	18,434,037 (43.13%)
Se2	54,194,598	48,463,529 (89.43%)	47,222,531 (87.14%)	1,240,998 (2.29%)	24,177,100 (44.61%)	24,187,190 (44.63%)
Se3	50,082,520	44,782,728 (89.42%)	43,671,155 (87.20%)	1,111,573 (2.22%)	22,351,691 (44.63%)	22,361,047 (44.65%)
SeMT1	53,746,644	46,764,890 (87.01%)	45,489,466 (84.64%)	1,275,424 (2.37%)	23,320,908 (43.39%)	23,341,541 (43.43%)
SeMT2	44,801,662	38,481,336 (85.89%)	37,476,825 (83.65%)	1,004,511 (2.24%)	19,193,707 (42.84%)	19,199,428 (42.85%)
SeMT3	53,976,028	46,688,836 (86.50%)	45,496,968 (84.29%)	1,191,868 (2.21%)	23,277,762 (43.13%)	23,312,249 (43.19%)

### 3.4 Functional classification of DEGs in grapevine

**Figure 4:**
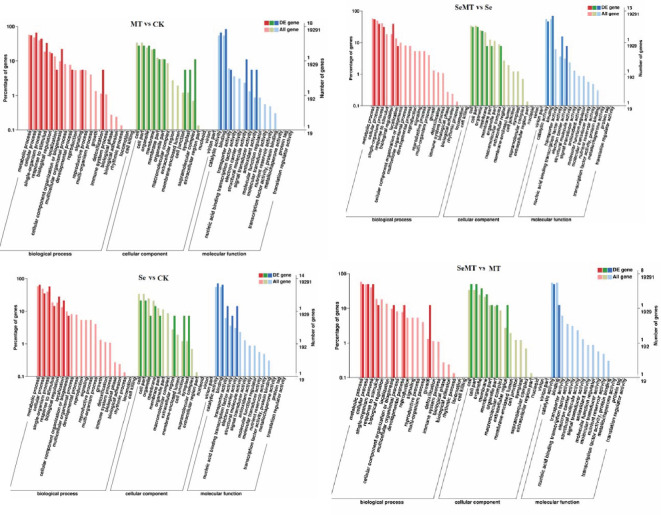
Go enrichment analysis of DEGs in grapevine.

**Figure 5:**
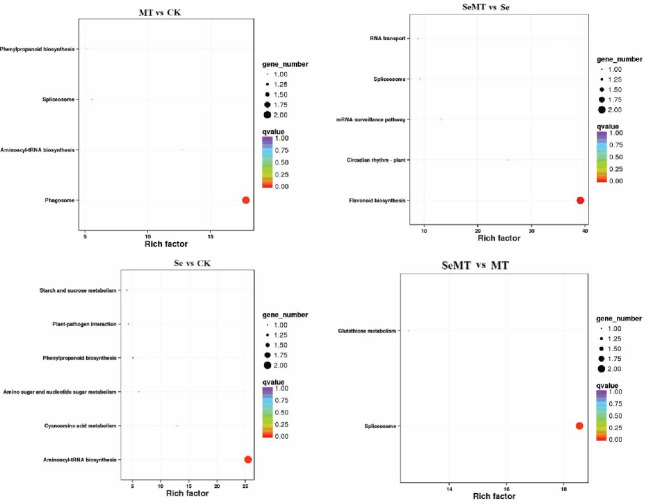
KEGG enrichment analysis of DEGs in grapevine.

The GO enrichment analyses showed that 18, 14, 13, and eight DEGs were enriched in the MT vs CK, Se vs CK, SeMT vs Se, and SeMT vs MT treatments, respectively (**Table 5**). There were three primary categories in GO annotation in the different comparison groups, including the biological process, cellular component, and molecular function (**Figure 4**). In the biological process, the GO annotation subcategories with higher gene frequencies included the metabolic process, cellular process, single-organism process, and response to stimulus. In the cellular component, the GO annotation subcategories with higher gene frequencies included the cell, cell part, organelle, and membrane. In the molecular function, the GO annotation subcategories with higher gene frequencies included the catalytic activity and binding. Subsequently, a KEGG pathway enrichment analysis detected nine, nine, eight, and four DEGs enriched in the MT vs CK, Se vs CK, SeMT vs Se, and SeMT vs MT treatments, respectively (**Table 5**). In the MT vs CK treatment, a total of five DEGs were annotated to four KEGG pathways, and only one pathway (phagosome) was enriched (**Figure 5**). In the Se vs CK treatment, a total of seven DEGs were annotated to six KEGG pathways, and only one pathway (aminoacyl-tRNA biosynthesis) was enriched. In the Se vs MT treatment, a total of two DEGs were annotated to two KEGG pathways, and only one pathway (spliceosome) was enriched. In the SeMT vs Se treatment, a total of six DEGs were annotated to five KEGG pathways, and only one pathway (flavonoid biosynthesis) was enriched. Moreover, there were nine DEGs related to Se metabolism that could be annotated with pathway information, involving nine metabolic pathways, including phenylpropanoid biosynthesis, aminoacyl-tRNA biosynthesis, cyanoamino acid metabolism, starch and sucrose metabolism, amino sugar and nucleotide sugar metabolism, plant-pathogen interaction, glutathione metabolism, flavonoid biosynthesis (ko00941), and flavonoid biosynthesis (ko00942) (**Table 6**).

**Table 5:** Statistics on the number of annotated DEGs in grapevine.

DEG Set	Total	COG	GO	KEGG	KOG	NR	Pfam	Swiss-Prot	EggNOG
MT vs CK	26	10	18	9	12	26	22	21	23
Se vs CK	17	9	14	9	14	17	15	15	16
SeMT vs Se	16	9	13	8	7	16	16	15	15
SeMT vs MT	10	4	8	4	7	10	9	8	9

**Table 6:** Functional classification of DEGs in grapevine.

Gene ID	Pathway and its ID	Enzyme and its ID
*VIT_13s0067g02360*	Phenylpropanoid biosynthesis: ko00940	Peroxidase (POD): K00430
*VIT_12s0035g01160*	Aminoacyl-tRNA biosynthesis: ko00970	Leucyl-tRNA synthetase: K01869
*VIT_06s0004g01430*	Cyanoamino acid metabolism: ko00460	β-glucosidase: K01188
	Phenylpropanoid biosynthesis: ko00940	
	Starch and sucrose metabolism: ko00500	
*VIT_15s0046g01570*	Amino sugar and nucleotide sugar metabolism: ko00520	Chitinase: K01183
*VIT_17s0000g02480*	Plant-pathogen interaction: ko04626	Calcium-binding protein CML: K13448
*VIT_19s0027g01190*	Aminoacyl-tRNA biosynthesis: ko00970	Phenylalanyl-tRNA synthetase α-chain: K01889
*VIT_19s0093g00220*	Glutathione metabolism: ko00480	Glutathione S-transferase: K00799
*VIT_00s0361g00040*	Flavonoid biosynthesis: ko00941	Anthocyanidin reductase: k21102
*VIT_05s0136g00260*	Flavonoid biosynthesis: ko00942	Chalcone synthase: K00660

### 3.5 Validation of the candidate DEGs in grapevine via qRT-PCR

**Figure 6:**
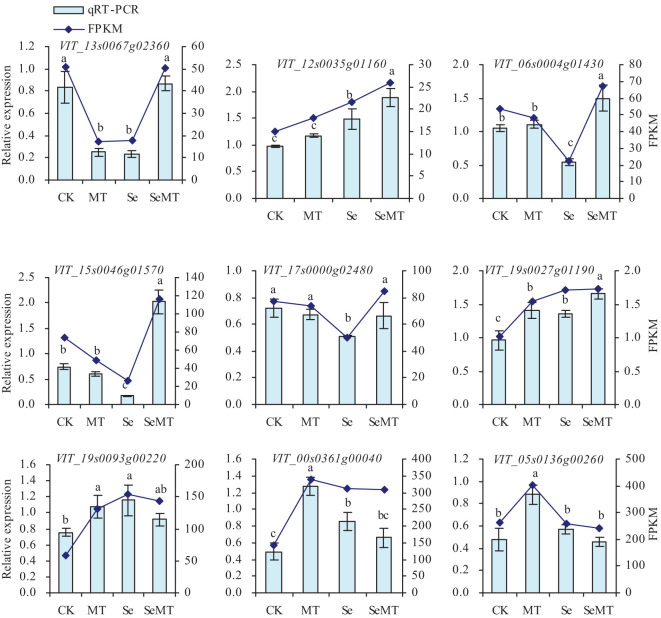
Relative expression levels of screened DEGs in grapevine. Values are means (± SE) of three replicates. Different letters indicate significant differences among the treatments (Duncan’s Multiple Range Test, *P* < 0.05).

To further verify the reliability of the trends of expression of the DEGs in the treatments of MT, Se, and SeMT, qRT-PCR was utilized in this study (**Figure 6**). The MT treatment downregulated the relative expression level of *VIT_13s0067g02360*, upregulated the relative expression levels of *VIT_19s0027g01190*, *VIT_19s0093g00220*, *VIT_00s0361g00040* and *VIT_05s0136g00260*, and had no significant effect on the relative expression levels of *VIT_12s0035g01160*, *VIT_06s0004g01430*, *VIT_15s0046g01570* and *VIT_17s0000g02480* under the treatments that lacked Se. Compared with CK, Se treatment downregulated the relative expression levels of *VIT_13s0067g02360*, *VIT_06s0004g01430*, *VIT_15s0046g01570* and *VIT_17s0000g02480*, upregulated the relative expression levels of *VIT_12s0035g01160*, *VIT_19s0027g01190*, *VIT_19s0093g00220* and *VIT_00s0361g00040*, and had no significant effect on the relative expression level of *VIT_05s0136g00260*. When the plants had been treated with Se, MT treatment upregulated the relative expression levels of *VIT_13s0067g02360*, *VIT_12s0035g01160*, *VIT_06s0004g01430*, *VIT_15s0046g01570*, *VIT_17s0000g02480* and *VIT_19s0027g01190*, and had no significant effect on the relative expression levels of *VIT_19s0093g00220*, *VIT_00s0361g0004*, and *VIT_05s0136g00260*. The nine DEGs that were screened also had similar trends of expression in the qRT-PCR and Fragments Per Kilobase per Million mapped fragments (FPKM).

### 3.6 Correlation, grey relational, PCA, and cluster analyses of grapevine parameters

**Figure 7:**
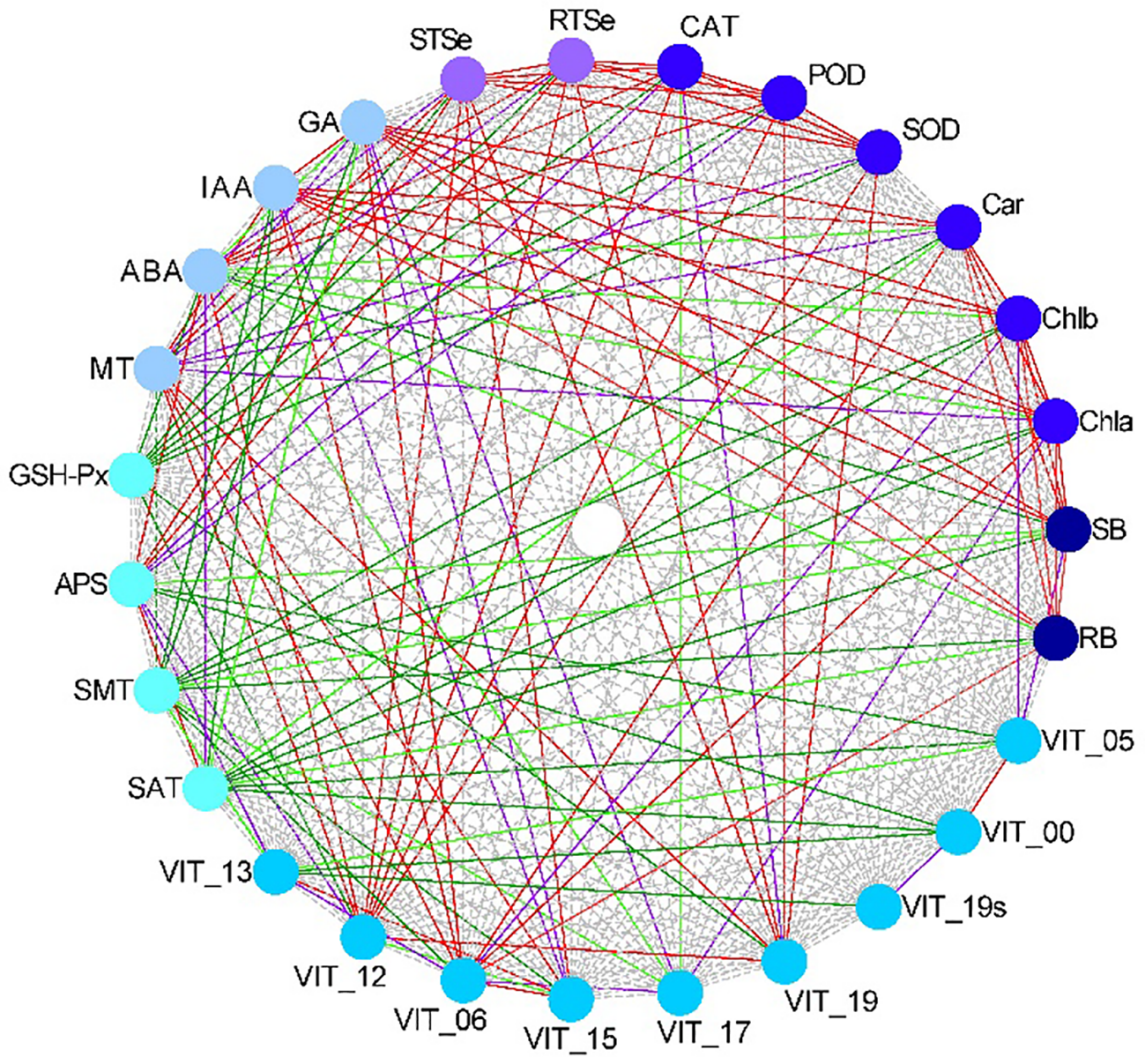
Correlations among different parameters in grapevine. The red solid line is highly significant positive correlation (*P* < 0.01), the purple solid line is significant positive correlation (0.01 ≤ *P* < 0.05), the dark green solid line is highly significant negative correlation (*P* < 0.01), and the light green solid line is significant negative correlation (0.01 ≤ *P* < 0.05). *N* = 12. RB, root biomass; SB, shoot biomass; Cha, chlorophyll *a* content; Chb, chlorophyll *b* content; Car, carotenoid content; SOD, SOD activity; POD, POD activity; CAT, CAT activity; RTSe, root total Se content; STSe, shoot total Se content; GA3, GA3 content; IAA, IAA content; ABA, ABA content; MT, MT content; GSHPx, GSH-Px activity; APS, APS reductase activity; SMT, SMT activity; SAT, SAT activity; VIT_13, relative expression level of *VIT_13s0067g02360*; VIT_12, relative expression level of *VIT_12s0035g01160*; VIT_06, relative expression level of *VIT_06s0004g01430*; VIT_15, relative expression level of *VIT_15s0046g01570*; VIT_17, relative expression level of *VIT_17s0000g02480*; VIT_19, relative expression level of *VIT_19s0027g01190*; VIT_19s, relative expression level of *VIT_19s0093g00220*; VIT_00 relative expression level of *VIT_00s0361g00040*; VIT_05, relative expression level of *VIT_05s0136g00260*.

**Figure 8:**
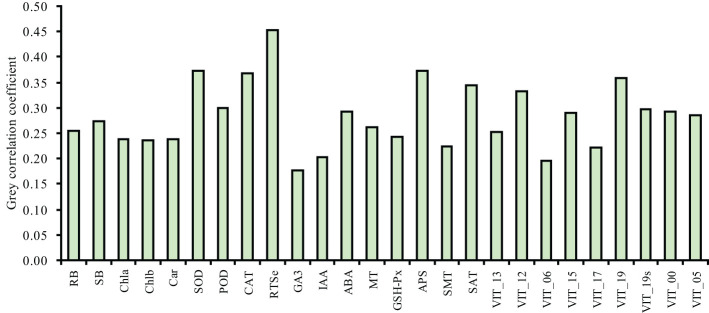
Grey correlation coefficients of the different parameters with the shoot total Se content in grapevine. RB, root biomass; SB, shoot biomass; Cha, chlorophyll *a* content; Chb, chlorophyll *b* content; Car, carotenoid content; SOD, SOD activity; POD, POD activity; CAT, CAT activity; RTSe, root total Se content; STSe, shoot total Se content; GA3, GA3 content; IAA, IAA content; ABA, ABA content; MT, MT content; GSHPx, GSH-Px activity; APS, APS reductase activity; SMT, SMT activity; SAT, SAT activity; VIT_13, relative expression level of *VIT_13s0067g02360*; VIT_12, relative expression level of *VIT_12s0035g01160*; VIT_06, relative expression level of *VIT_06s0004g01430*; VIT_15, relative expression level of *VIT_15s0046g01570*; VIT_17, relative expression level of *VIT_17s0000g02480*; VIT_19, relative expression level of *VIT_19s0027g01190*; VIT_19s, relative expression level of *VIT_19s0093g00220*; VIT_00 relative expression level of *VIT_00s0361g00040*; VIT_05, relative expression level of *VIT_05s0136g00260*.

**Figure 9:**
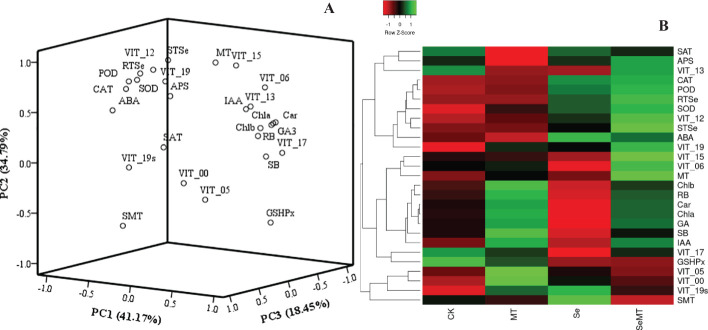
PCA and cluster heatmap of different parameters in grapevine. **(A)** PCA; **(B)** cluster heatmap. RB, root biomass; SB, shoot biomass; Cha, chlorophyll *a* content; Chb, chlorophyll *b* content; Car, carotenoid content; SOD, SOD activity; POD, POD activity; CAT, CAT activity; RTSe, root total Se content; STSe, shoot total Se content; GA3, GA3 content; IAA, IAA content; ABA, ABA content; MT, MT content; GSHPx, GSH-Px activity; APS, APS reductase activity; SMT, SMT activity; SAT, SAT activity; VIT_13, relative expression level of *VIT_13s0067g02360*; VIT_12, relative expression level of *VIT_12s0035g01160*; VIT_06, relative expression level of *VIT_06s0004g01430*; VIT_15, relative expression level of *VIT_15s0046g01570*; VIT_17, relative expression level of *VIT_17s0000g02480*; VIT_19, relative expression level of *VIT_19s0027g01190*; VIT_19s, relative expression level of *VIT_19s0093g00220*; VIT_00 relative expression level of *VIT_00s0361g00040*; VIT_05, relative expression level of *VIT_05s0136g00260*.

To analyze the relationships of the different parameters with the content of total Se in the shoot, correlation, grey relational, PCA and cluster analyses were used in this study. The correlation analysis showed that the content of total Se in shoot positively correlated with the SOD activity, POD activity, CAT activity, APS reductase activity, root total Se content, ABA content, MT content, and the relative expression levels of *VIT_12s0035g01160*, *VIT_15s0046g01570*, and *VIT_19s0027g01190*, and negatively correlated with the activity of GSH-Px (**Figure 7**). The grey relational analysis showed that the content of total Se in shoot correlated with all the other parameters (**Figure 8**). The grey correlation coefficients of root total Se content and the activities of APS reductase, SOD and CAT, and relative expression levels of *VIT_19s0027g01190* > 0.35, which were the top five parameters closely associated with the content of total Se in shoots. In addition, the PCA and cluster analysis also showed that the contents of total Se in shoots and roots, the activities of APS reductase, SOD and CAT, and relative expression levels of *VIT_19s0027g01190* clustered in a category (**Figures 9A, B**).

### 3.7 Quality and total content of Se in grapes

When the plants had not been treated with Se, MT treatment increased the single fruit weight and the contents of total soluble solids, total soluble solid content/titratable acid content, and vitamin C and decreased the titratable acid content of grapes (**Table 7**). Compared with CK, Se treatment also increased the single weight and the contents of total soluble solids and vitamin C and the total soluble solid content/titratable acid content, and decreased the titratable acid content of grapes. When the plants had been treated with Se, the SeMT treatment increased the contents of total soluble solids and vitamin C and the total soluble solid content/titratable acid content, decreased the content of titratable acid, and had no significant effect on the weight of single grapes. In addition, MT treatment increased the content of Se in grapes treated with and not treated with Se. The order of total Se content in grapes was SeMT treatment > Se treatment > MT treatment > CK (**Table 5**).

**Table 7:** Quality and Se content of grapes.

Treatment	Single fruit weight (g)	Total soluble solid content (%)	Titratable acid content (%)	Total soluble solid content/titratable acid content	Vitamin C content (mg/100mg FW	Total Se content (mg/kg DW)
CK	2.363 ± 0.029c	16.5 ± 0.15d	0.373 ± 0.009a	44.30 ± 0.20d	1.917 ± 0.178c	0.021 ± 0.002d
MT	2.512 ± 0.019b	17.7 ± 0.23c	0.312 ± 0.025c	56.82 ± 0.81b	2.582 ± 0.126b	0.029 ± 0.001c
Se	2.630 ± 0020a	18.6 ± 0.15b	0.352 ± 0.007b	52.84 ± 0.14c	2.490 ± 0.160b	0.127 ± 0.005b
SeMT	2.662 ± 0.025a	20.9 ± 0.10a	0.302 ± 0.016c	69.21 ± 0.76a	3.459 ± 0.072a	0.174 ± 0.006a

Values are means (± SE) of three replicates. Different letters indicate significant differences among the treatments (Duncan’s Multiple Range Test, P < 0.05). DW, dry weight; FW, fresh weight.

## 4 Discussion

### 4.1 Effects of MT on the growth and physiology of grapevines under Se stress

When plants are in external environmental stresses, it severely inhibits their growth and development (Zheng et al., 2009). Suitable concentrations of Se can increase energy metabolism and promote the growth of plants, but high concentrations of Se can be toxic to plants and inhibit their growth (Ramos et al., 2010). In this study, Se treatment with decreased the biomass and the contents of photosynthetic pigments, GA3, and IAA of grapevines and increased the activities of SOD, POD, and CAT, and the content of ABA (**Figures 1A–G**), which indicates that Se treatment inhibited the growth of grapevines. These results are consistent with the results of previous studies (Peng et al., 2020; Wang et al., 2024), which further indicates that high levels of Se cause significant amounts of ROS to accumulate (Zhang et al., 2016), damage the chloroplasts, and inhibit the biosynthesis of photosynthetic pigments (Saffaryazdi et al., 2012; Lehotai et al., 2016), and alter the levels of phytohormones, which results in a hormonal homeostasis imbalance in the plants (Jiang et al., 2019; Malheiros et al., 2019). This combination of factors inhibits the growth of plants.

Studies have shown that MT can increase the tolerance of plants to toxic compounds and heavy metals (Tan et al., 2006). Under stress conditions, MT increases the biomass of soybean (Zhang et al., 2021), inhibits the degradation of photosynthetic pigments, improves the stability of photosynthetic pigments (Ye et al., 2015; Liu et al., 2019), increases the activities of antioxidant enzymes, and inhibits the rate of production of superoxide anions (O_2_^-^) and hydrogen peroxide (H_2_O_2_) (Zhou et al., 2020; He et al., 2022). In this study, MT increased the biomass, contents of photosynthetic pigments, and the activities of SOD, POD, and CAT of grapevine with and without Se stress (**Figures 1A–E**), which indicates that MT can promote the growth of grapevine and alleviate Se stress. These results may be related to the inhibitory effects of MT on the key enzyme of chlorophyll degradation (demagnesyl chlorophyll *a* oxygenase) genes and the transcript level of the gene *SAG12* that is related to leaf senescence (Wang et al., 2012) and the effects of MT at enhancing the levels of activity of antioxidant enzymes to scavenge excess ROS in plant tissues, which promotes the growth of plants (Ulhassan et al., 2019).

Moreover, the application of MT helps to increase the content of free IAA in the plant roots (Chen et al., 2019), which suggests a reciprocal effect between MT and IAA. MT also improves the drought tolerance of plants by downregulating the ABA biosynthetic gene *MdNCED3* and upregulates its catabolic genes *MdCYP707A1* and *MdCYP707A2*, thereby reducing the content of ABA in plants (Li et al., 2015). In addition, MT upregulates the GA3 biosynthetic genes, which results in an increase in the content of GA3 in cucumber, thus, is alleviating the stress on its growth (Zhang et al., 2014). In this study, MT increased the contents of GA3, IAA, and MT, while it decreased the content of ABA in grapevines treated with or without Se (**Figures 1F, G**), which is consistent with the results of previous studies (Zhang et al., 2014; Li et al., 2015; Chen et al., 2019). MT has effects that are similar to those of IAA in regulating plant growth (Ruiz et al., 2005). GA3, IAA, and MT are related to the growth of plants, and their higher contents in plants result in quicker growth (Yang et al., 2013). ABA can inhibit plant growth and accelerate plant senescence (Yuan et al., 2014). Thus, MT could improve plant tolerance to stress by regulating the balance of endogenous hormones in plants.

### 4.2 Effects of MT on the accumulation of Se by grapevines

Selenite that is taken up by plants is converted to selenide, which is converted to selenocysteine by OAS-TL and SAT (Wallenberg et al., 2010; Yarmolinsky et al., 2013). The increase in Se in the environment promotes the levels of expression of *SAT*, which, in turn, enhance the conversion of selenocysteine (Van Hoewyk et al., 2008). The toxic effect of Se on plants is primarily reflected in the ability of SeCys and SeMet to replace the normal amino acids in proteins, which results in alterations in the structure and activity of proteins. The ability of plants to methylate SeCys and SeMet to MeSeCys and methyl SeMet via SMT and methionine methyltransferase (MMT), thus, avoids their involvement in protein structures (van Hoewyk, 2013; Chen et al., 2020). In this study, Se stress increased the activity of SMT and decreased that of GSH-Px, while it had no significant effect on the activities of APS reductase and SAT of grapevine (**Figures 2E, F**). Thus, the uptake of Se in grapevine may be a passive state under Se stress, and SMT plays an important role in alleviating the toxicity of Se by converting selenide to SeCys (Sors et al., 2009). In addition, MT decreased the activities of GSH-Px, APS reductase, SMT, and SAT when there was a lack of Se stress (**Figures 2E, F**). Under Se stress, MT increased the activity of APS reductase, decreased that of SMT, and had insignificant effects on the levels of GSH-Px and SAT activity. MT increased the content of Se in grapevine and the ratio of organic Se to total Se in the shoots and had no significant effect on the TF of grapevine (**Table 2**; **Figures 2A–D**). These results are consistent with the findings of previous studies (Liao et al., 2018; Cheng et al., 2022), which indicate that MT may alleviate the toxicity of Se to grapevine, and promote the conversion of inorganic Se to organic Se. Moreover, the content of total Se in grapevine roots was higher than that of the shoots, and the content of organic Se was higher than the content of inorganic Se in the roots and shoots of grapevine (**Table 2**; **Figures 2A, C, D**). These results indicate that the accumulation of Se in grapevine is primarily concentrated in the roots. The Se that is absorbed by the roots is converted into SeCys or SeMet, which is easily immobilized in roots, and only a small portion is transported to the aboveground parts through the xylem (Li et al., 2008). The transcriptome sequencing analysis (**Table 5**; **Figure 4**) showed that the categories of GO annotations with higher frequency of differences when MT was or was applied were as follows: cellular processes, metabolic processes, and catalytic activity binding among others that indicates that MT could respond to Se stress via the regulation of multiple functional genes. The KEGG enrichment analysis (**Table 5**; **Figure 5**) showed that a total of nine DEGs related to Se metabolism were annotated with pathway information, and these nine DEGs also had similar trends of gene expression in the qRT-PCR and FPKM (**Figure 6**). These nine DEGs (**Table 6**) involved nine metabolic pathways, including the phenylpropanoid biosynthesis, aminoacyl-tRNA biosynthesis, cyanoamino acid metabolism, starch and sucrose metabolism, amino sugar and nucleotide sugar metabolism, plant-pathogen interaction, glutathione metabolism, flavonoid biosynthesis (ko00941), and flavonoid biosynthesis (ko00942).

Among these differentially expressed pathways, the gene *VIT_12s0035g01160* that is related to aminoacyl-tRNA biosynthesis and cyanoamino acid metabolism and the gene *VIT_19s0027g01190* that is related to aminoacyl-tRNA biosynthesis were upregulated by MT under Se stress (**Figure 6**), which could lead to the upregulated levels of expression of leucyl-tRNA synthetase and phenylalanyl-tRNA synthetase α-chain and increase the biosynthesis of valine, leucine, and isoleucine (Li, 2021). The gene *VIT_19s0093g00220* that is related glutathione metabolism was upregulated following treatment with MT, Se, or MT + Se (**Figure 6**). The glutathione metabolic pathway that involves glutathione S-transferase can participate in the resistance of plants to oxidative stress and scavenge endogenous deleterious compounds in plants and upregulates the expression of glutathione S-transferase, which has been recognized as one of the important markers of corresponding stresses in plants (Chen et al., 2004).

Previous studies have shown that MT enriched the DEGs of cotton seedlings in pathways, such as phenylalanine synthesis and phytohormone signaling, under salt stress (Chen, 2020). In the dark, MT downregulates the expression of genes of the pathways related to the biosynthesis of flavonoids, which suggests that MT inhibits their biosynthesis, and thus, alleviates the yellowing of gardenia leaves (Wang et al., 2018). In this study, the expression of proteins related to plant-pathogen interaction (the related gene *VIT_17s0000g02480*) was downregulated by treatment with Se (Se stress) and upregulated by MT under Se stress (**Figure 6**). The expression of chalcone synthase, a key enzyme in the biosynthesis of flavonoids, had a downregulated trend of expression in the pathway of flavonoid biosynthesis (the related genes *VIT_00s0361g00040* and *VIT_05s0136g00260*) under Se stress following treatment with MT. These results indicate that MT could help to delay the senescence of grapevine leaves under Se stress, which is consistent with the results of a previous study (Wang et al., 2018). In this study, the metabolic pathways that differed significantly between the treatments were primarily metabolic and bioregulatory processes, and the primary enzymes involved were POD (the related gene *VIT_13s0067g02360*), β-glucosidase (the related gene *VIT_06s0004g01430*), and chitinase (the related gene *VIT_15s0046g01570*) (**Figure 6**). The levels of expression of these related genes were downregulated by Se treatment (Se stress) and upregulated by MT under Se stress. These results indicate that MT could help to clear the accumulation of toxic compounds in grapevine owing to stress conditions. In addition, the correlation, grey relational, PCA, and cluster analyses (**Figures 7**-**9**) showed that the content of total Se in the roots, the activities of APS reductase, SOD, and CAT, and the levels of expression of *VIT_19s0027g01190* were closely associated with the content of total in the shoots, which highlights their significant role in promoting the uptake of Se in grapevine under Se stress.

### 4.3 Effect of MT on the quality of grapes and their accumulation of Se

Studies have shown that MT can increase the single weights of pears and peaches (Liu, 2019; Wu et al., 2021). MT promotes the growth and expansion of grapes and makes them uniform in size (92). In this study, MT increased the single weight of grapes when the plants had not been treated with Se (**Table 7**), which is consistent with the findings of previous studies (Meng et al., 2015; Liu, 2019; Wu et al., 2021). This indicates that MT can promote the growth of grapes. This result may be related to the similar role of MT to IAA in regulating plant growth (Ruiz et al., 2005). However, MT had no significant effects on the single weight of grapes that had been treated with Se. Se can promote the growth of grapes and increase their single weight (Dou et al., 2021). The plants were only treated with MT + Se for a short period of time in this study, thus, the overlapping effects of MT and Se were not readily apparent. In addition, MT also increases the contents of total soluble solids, soluble sugar, and vitamin C in fruits (Zhang et al., 2019; Huang et al., 2020; Wu et al., 2021), increases the content of titratable acid in cherry tomatoes and apricots (Zhang et al., 2019; Huang et al., 2020), while it decreases the titratable acid content in peaches and grapes (Wu et al., 2021; Xia et al., 2021). In this study, MT increased the contents of total soluble solids and vitamin C, increased the content of total soluble solid content/titratable acid, and decreased the titratable acid content of grapes (**Table 7**). These findings are consistent with those of previous studies (Wang et al., 2021; Wu et al., 2021; Xia et al., 2021) and may be attributed to the fact that MT delays the senescence of leaves, promotes photosynthesis, and increases the accumulation of sugars in fruits (Zhang et al., 2016).

Selenocysteine, sodium selenite, and sodium selenate can induce the biosynthesis of MT in tomato (Li et al., 2016), which indicates that Se is closely related to MT. However, the treatment of MT + Se inhibits the uptake of Se in tomatoes under Cd stress, and only the treatment with Se promotes its uptake (Zang, 2020). In this study, MT increased the content of Se in grapes that were treated and not treated with Se, which indicates that MT can promote the accumulation of Se in grapes (**Table 7**). This result differs from the findings of Zang (2020), which may be related to the different stress conditions.

## 5 Conclusions

Under Se stress, exogenous MT promoted the growth, regulated the nutrient uptake and hormone balance, and improved the resistance of grapevine, and it also promoted the uptake of Se and the conversion of inorganic Se to organic Se in grapevine. Exogenous MT primarily regulated aminoacyl-tRNA biosynthesis, spliceosome and flavonoid biosynthesis, which involved a total of nine DEGs and nine metabolic pathways, to promote the growth and uptake of Se by grapevines when stressed by Se. In addition, exogenous MT increased the content of Se and improved the quality of grapes. Therefore, MT can be used to enrich Se during the production of grapes.

## Data Availability

The raw sequence data reported in this paper have been deposited in the National Center for Biotechnology Information (NCBI) and are publicly accessible at https://www.ncbi.nlm.nih.gov/sra/PRJNA1134381.
